# ‘It’s always difficult when it’s family. . . whereas when you’re talking to a therapist. . .’: Parents’ views of cognitive-behaviour therapy for depressed adolescents

**DOI:** 10.1177/13591045211013846

**Published:** 2021-05-19

**Authors:** Katharina Schlimm, Maria Loades, Emily Hards, Shirley Reynolds, Monika Parkinson, Nick Midgley

**Affiliations:** 1 Department of Psychology, University of Bath, UK; 2 Bristol Medical School, University of Bristol, UK; 3 School of Psychology and Clinical Language Studies, University of Reading, UK; 4 Research Department of Clinical, Educational and Health Psychology, University College London, UK; 5 Child Attachment and Psychological Therapies Research Unit (ChAPTRe), Anna Freud National Centre for Children and Families, London, UK

**Keywords:** Depression, cognitive-behavioural therapy, CBT, adolescent, parent, qualitative, RCT

## Abstract

**Background:**

Parents are key to helping their adolescent child access psychological therapy for mental health problems such as depression. However, little is known about how parents experience their child’s psychological therapy. We aimed to explore parents’ experiences of their adolescent child’s cognitive behaviour therapy for depression.

**Method:**

We applied Thematic Analysis (TA) to qualitative data from in-depth interviews with parents (*N* = 16) whose adolescent child was randomly allocated to CBT in a large multisite RCT for adolescent depression (the IMPACT trial). Interviews were conducted at the end of treatment.

**Results:**

We generated two main themes: parents’ perceptions of the adolescent’s journey through therapy, and parents’ perceptions of the therapeutic setting and process. Each included four sub-themes. Parents talked about key factors that impacted on their child’s progress through treatment, including the adolescent’s readiness for therapy and the adolescent-therapist relationship.

**Conclusion:**

Parents’ insights confirm the foundations of what is considered good clinical practice of CBT for adolescent depression, including tailoring therapy to the adolescent, and establishing a strong adolescent-therapist relationship. Parents recognised that, for CBT to be helpful, their child had to be willing to engage in therapy and able to develop a trusting relationship with their therapist.

Depression is one of the most common mental problems, with 121 million individuals worldwide experiencing depression at some stage in their life (Steinberg & Daniel, 2020). Depression is also the leading cause of disability-adjusted life years lost among 10 to 19-year-olds (Lu, 2019). In England, the point prevalence of depression was 2.7% among 11 to 16-year-olds and 4.8% among 17 to 19-year-olds, with higher rates among females than males (National Health Service [NHS], 2018).

Depression negatively impacts on adolescents’ social interactions and sense of identity and is frequently accompanied by feelings of worthlessness (Shaw et al., 2009). It is associated with cognitive and functional impairment (Hammer-Helmich et al., 2018; Snyder, 2013), substance abuse (Grant et al., 2016) and increased risk of suicide (Akil et al., 2018). Depressed young people often have other mental health disorders including anxiety and eating disorders (Avenevoli et al., 2001). Risk factors for adolescent depression include negative school experiences as well as overprotective, controlling parenting styles and low levels of nurturance and affection (Betts et al., 2009).

Evidence for the effectiveness of CBT for adolescent depression has been somewhat varied and it is, at best, moderately effective (Weersing et al., 2017; Weisz et al., 2017). In the largest existing trial of psychological treatments for adolescent depression, CBT was equally effective in reducing depression symptoms, although it did not outperform the two comparators, brief psychosocial intervention and short-term psychoanalytic therapy (Goodyer et al., 2017a). CBT has also been compared to and combined with medication, for example, in the Treatment of Adolescents with Depression Study (TADS). At 12 weeks post-randomisation, the combination of CBT plus fluoxetine was more effective in reducing depression than CBT alone, and fluoxetine alone also outperformed CBT alone (March et al., 2004). Factors such as age and severity predicted 12-week treatment outcomes in TADS; younger adolescents, those who were less severely affected and impacted by depression, those with fewer co-morbid diagnoses, and those with greater expectations for treatment were more likely to benefit (Curry et al., 2006). At 36 weeks post-randomisation, the response rates for all three active treatment conditions were >80% (March et al., 2007).

Research has consistently identified that parents of a child with mental health difficulties frequently experience distress, including feelings of grief, fear and guilt (Armitage et al., 2020; Mohr & Regan-Kubinski, 2001). Many parents fear their child self-harming or exhibiting suicidal behaviours (Armitage et al., 2020). Parents of depressed adolescents typically express helplessness and devote much of their time and energy to their child’s difficulties (Stapley et al., 2016). Many report feelings of loss in relation to their child’s personality, societal and familial role and joy in life (MacGregor, 1994). Furthermore, seeing their child struggle can make parents question their competence as parents and alter family dynamics, beliefs and self-perceptions (MacGregor, 1994). Most parents of adolescent children who experience mental health difficulties were found to blame themselves partially or fully for their child’s difficulties (Moses, 2010). Reasons for self-blame included perceived bad parenting, hereditary transmission and negative family environment. Studies have also found that parents of depressed adolescents reported lower levels of well-being than parents of non-depressed adolescents (Early et al., 2002; Perloe et al., 2014).

Parents may be involved in CBT for adolescents in various ways including as co-therapists, co-clients and supporters of therapy (Stallard, 2002). The nature of parent involvement in CBT for adolescent depression tends to depend on the needs of the individual adolescent and their family and may change over the course of therapy (Verduyn et al., 2009). A systematic review found that CBT including joint adolescent-parent sessions was more successful at reducing depressive symptoms than individual therapy with the adolescent alone (Dardas et al., 2018). Interventions involving parent-adolescent joint sessions led to more positive outcomes than separate parent sessions by facilitating parent-adolescent interaction and positive parenting. There was evidence that joint sessions positively impacted on the parent-child relationship and reduced family conflict, and joint sessions resulted in superior outcomes at two-year follow-up (Dardas et al., 2018). These findings support earlier research (Gilham et al., 2006/; McCarty et al., 2013), which found that CBT involving parents significantly reduced adolescents’ depressive symptoms.

Whether or not parents are actively involved in CBT, parents frequently play a crucial role in helping their adolescent child seek out therapy and they tend to facilitate continued attendance. Therefore, their perceptions of their child’s therapy may influence engagement in therapy. Disengagement from therapy is a particular problem in adolescents who are depressed, possibly due to amotivation and hopelessness (O’Keeffe et al., 2019). For example, the dropout rate in the IMPACT trial was relatively high (37%; O’Keeffe et al., 2019). Exploring parents’ experiences of psychological therapy for depression for their adolescent child may provide valuable information about continued engagement in therapy.

Previous qualitative studies have explored the experience of being a parent of a depressed adolescent (Stapley et al., 2016) and parents’ experience of their child’s treatment following a self-harm episode (Stewart et al., 2016). However, despite the evidence of the impact of adolescent depression on family members and the role of parents in CBT for adolescent depression, little is known about how parents experience their adolescent child having treatment for depression. We aimed to fill this gap by qualitatively exploring parents’ experiences of their adolescent child’s CBT for depression. The qualitative approach was considered most appropriate for gathering in-depth and rich personal accounts to address this gap. Exploring parents’ experiences may provide novel insights to help clinicians tailor therapy more closely to adolescents’ needs, potentially enhancing treatment outcomes and helping parents feel more engaged and supported.

## Method

### Study design

We analysed data from IMPACT-My Experience (IMPACT-ME; Midgley et al., 2014), a qualitative study that was nested within the Improving Mood with Psychoanalytic and Cognitive Therapies (IMPACT) study (Goodyer et al., 2011), a large multi-centre randomised controlled trial (RCT). The IMPACT trial compared the effectiveness of three psychological therapies for adolescent depression. For details see Goodyer et al. (2011) and Goodyer et al. (2017a, 2017b). In summary, participants were aged 11 to 17 and met diagnostic criteria for moderate to severe depression as classified by Diagnostic and Statistical Manual for Mental Disorders (DSM-IV) (American Psychiatric Association, 1994).

The IMPACT-ME study (Midgley et al., 2014) provided a longitudinal exploration of how the three randomly allocated treatments were experienced by adolescents, their parents and therapists. Adolescents and parents were interviewed at three different time points: before therapy (Time 1; baseline), after therapy (Time 2; 36 weeks after baseline) and 12 months later (Time 3). IMPACT-ME was conducted within the North London arm of the IMPACT RCT. Therefore, only those IMPACT study participants from this geographical area were recruited to the qualitative sub-study (*N* = 77). Of these, 27 adolescents had been randomised to CBT. Parents of 18 of these participants agreed to take part in the IMPACT-ME study and were interviewed after their son or daughter ended therapy. However, we excluded two further participants as their adolescent child did not start IMPACT study CBT.

In the IMPACT trial, CBT was carried out over the course of 28 weeks and comprised up to 20 sessions. There was variability in how much parents were involved in treatment. Some parents were not involved at all in their child’s treatment, others were present in the first half of the adolescent’s session or the last ten minutes or attended joint parent-adolescent sessions. Data was not collected on the extent to which parents were involved in each instance.

The present study aimed to capture parents’ experiences and perceptions of their child’s CBT. To address our aim, we focused on Time 2 (end of therapy) interviews from the IMPACT-ME study with parents whose child was assigned to CBT. This criterion yielded 16 interviews (14 mothers and two fathers, adolescent mean age = 15.6, *SD* = 1.27). For participant demographics, see Table 1.

**Table 1.** Participants’ demographic details.

Parent pseudonym	Parent’s gender	Child’s gender	Child’s age (years)	Child’s ethnic origin	Parent has completed further education? (Number of years)	Approximate family income (£)	Disengaged from treatment prematurely? (i.e. dropouts or treatment completers)
Susan	Female	Female	17	Mixed	Yes, 4 years	Missing	Dropout
Felicity	Female	Female	16	White	No	<10,000	Completer
Anne-Marie	Female	Male	16	Mixed	Yes, 4 years	10,000–20,000	Completer
Karen	Female	Female	16	White	No	75,000–100,000	Completer
Jane	Female	Male	16	White	Yes, 3 years	50,000–75,000	Completer
Melanie	Female	Female	13	White	Yes, 2 years	10,000–20,000	Completer
Helen	Female	Male	15	Mixed	Yes, 6 years	50,000–75,000	Completer
Joanne	Female	Male	14	Mixed	No	Missing	Dropout
Julie	Female	Female	15	White	No	Missing	Completer
Diane	Female	Male	17	White	No	Missing	Completer
Carol	Female	Female	14	Black/Black British	Yes, 4 years	<10,000	Completer
Mike	Male	Female	17	White	Yes, 3 years	20,000–30,000	Completer
Phil	Male	Female	15	Black/Black British	Yes, 5 years	10,000–20,000	Completer
Lynne	Female	Male	15	White	Yes, 2 years	<10,000	Completer
Nancy	Female	Male	18	White	Yes, 4 years	75,000–100,000	Dropout
Sophie	Female	Female	15	White	Yes, 4 years	30,000–50,000	Dropout

### Ethical considerations

Ethical approval for the IMPACT and IMPACT-ME studies was given by the Cambridgeshire two Research Ethics Committee (reference 09/H0308/137) and local NHS provider trusts. The data for the IMPACT-ME is held and owned by the Anna Freud Centre. The Psychology Research Ethics Committee at the University of Bath also granted ethical approval of the present study.

### Data collection

The interviews for the IMPACT-ME study were conducted by trained research assistants, using the semi-structured ‘Experience of Therapy’ interview topic guide (Midgley et al., 2011). Research assistants were post-graduate psychologists who received training from the IMPACT-ME research team in conducting qualitative, semi-structured interviews and in using the interview schedules and were closely supervised (Midgley et al., 2016; Stapley et al., 2017).

Interviews were conducted either at the participant’s home or at the clinic where treatment was delivered, depending on parents’ preferences. Interviews explored parents’ perceptions of the adolescents’ experience of therapy, changes over time and factors resulting in positive or negative outcomes (Midgley et al., 2014). Topics explored in the interview included: the parent’s impressions of the adolescent’s therapist: any changes in the adolescent since first being referred for help, how the parents understood what contributed to those changes (within or outside therapy); and what parents valued about therapy or wished had different. Interviews were audio-recorded and transcribed verbatim. IMPACT-ME participants were given a pseudonym and any individual-specific data was removed or anonymised in the transcripts to ensure confidentiality.

### Data analysis

Thematic Analysis (TA) was used to analyse the data (Braun & Clarke, 2006; Braun et al., 2014). This qualitative method was chosen because it enables the researcher to acquire a rich and detailed understanding of the phenomenon being investigated. An inductive, realist approach to TA was adopted, meaning that themes were identified based on participants’ accounts of their experiences and how they make meaning of these (Braun & Clarke, 2006) rather than looking for pre-determined categories.

Data analysis followed six stages as recommended by Braun and Clarke (2006, 2012). First, the primary researcher [KS] familiarised herself with the data by reading the transcribed interviews repeatedly and taking initial notes before systematically coding the entire data set. To ensure reliability of the analysis (Boyatzis, 1998) and that the codes identified by the primary researcher reflected the data, other researchers [GH and JM] examined 11% of the un-coded transcripts and gave feedback. To minimise researcher bias, KS, GH and JM met regularly to discuss and reflect on findings with supervisory oversight [ML, EH]. Second, the primary researcher generated potential themes, collating all data relevant for each theme. Next, the themes were reviewed using a thematic map to ensure they were consistent with the coded extracts and with the data. The themes were then defined, refined, and named, with modifications being made where necessary. Lastly, the themes were reported and interpreted using illustrative quotes.

## Results

Two main themes were identified from a thematic analysis of the 16 interviews (Table 2).

**Table 2.** Themes and sub-themes.

Themes	Subthemes
1. Parental perceptions of the adolescent’s journey through therapy	1. Willingness to engage in therapy
	2. Respecting the adolescent’s privacy and offering control
	3. Re-emergence of old personality as symptoms recede
	4. Lessons learned from CBT
2. Parental perceptions of the therapeutic setting and process	1. The importance of a positive patient-therapist relationship
	2. Parents’ trust and confidence in the therapist
	3. Lack of communication with parents
	4. Perceived advantages and disadvantages of parental involvement

### Theme 1. Parental perceptions of the adolescent’s journey through therapy

This theme captures how parents perceived their adolescent child’s progress through different stages of therapy. Some young people ended therapy prematurely. The following subthemes were identified: willingness to engage in therapy, respecting the adolescent’s privacy and offering control, re-emergence of old personality as symptoms recede, and lessons learned from CBT.

#### Willingness to engage in therapy

Parents talked about whether the adolescent was ready for therapy. Some described their child as being willing to engage in therapy:

*She kept saying to me ‘I want to get better’. . . she realised that she needed the help. (Karen)*

Adolescents who wanted therapy typically were seen by their parents as having made positive change. By contrast, those who parents believed were not ready to accept help were felt to have made little to no change. One parent viewed the adolescent’s conviction that therapy would be unsuccessful as likely to have resulted in a self-fulfilling prophecy:

*She’d decided from that first session that it wasn’t going to work. . . (Felicity)*

Felicity also described her daughter displaying behaviour which she framed as an indication that her daughter was not ready for therapy, noting *‘she sat there pulling stupid faces and behaving like a three year old’*. Her daughter’s behaviour may indicate defiance, or a lack of willingness and motivation to engage in therapy, possibly due to feelings of hopelessness or pessimism regarding the outcome of therapy.

Another sign of unwillingness was parents having to force adolescents to attend sessions. Joanne stated she *‘dragged’* her son. Similarly, Susan remarked she had to *‘make her [the adolescent] go’*. Furthermore, unwilling adolescents didn’t follow through on tasks agreed in therapy:

*He didn’t do the CBT work that she’d given him to do. . . (Helen)*

Adolescents who were unwilling to engage in therapy often ended therapy prematurely, with Joanne noting her son went *‘to maybe three [sessions] at the max’.*

#### Respecting the adolescent’s privacy and offering control

Most parents tended not to ask their son or daughter questions about therapy, instead letting their child decide what information they felt like sharing. Jane stated that her son *‘*sometimes . . . tells me what they’ve talked about and sometimes he doesn’t’.

Many parents did not want to appear intrusive:

*I made a point of never. . . badgering her to find out what happened in the session. . . if she wanted to tell me anything she would. . . (Melanie)*

Parents also addressed the importance of their child regaining a sense of control by letting the adolescent decide on parental involvement in therapy. Mike explained *‘we gave (name of adolescent) the choice’.* Jane indicated that her son assumed an active role in his journey through therapy:

*It’s the control that (name of adolescent) has over his own treatment. . . he’s not being swept along on this tide of interventions. . . (Jane)*

Parents perceived control as crucial for facilitating positive outcomes. Jane stated that *‘giving him that choice is given him more confidence’*, because *‘at his lowest he didn’t feel he had a choice’*. Lynne noted that making even small decisions throughout therapy – for example, choosing the room for sessions – made her son feel more in control.

#### Re-emergence of old personality as symptoms recede

Parents frequently described the adolescent’s ‘old personality’ re-emerging as depressive symptoms reduced. One parent seemed taken by surprise when her daughter exhibited a pre-depression behaviour:

*One day. . . she just started singing in the car and I hadn’t heard that for. . . over a year. . . (Karen)*

Most parents who saw their child’s treatment as beneficial portrayed the adolescent as returning to their old self. Mike noted *‘she’s back to her normal self now’.* Helen stated *‘he’s back’*, suggesting that she felt like the depression had temporarily taken over her son’s personality. Furthermore, parents described the adolescent’s sense of humour returning as depressive symptoms eased. Mike noted *‘she stopped joking with us. . . now that’s all back. . . with a vengeance’*.

Many parents witnessed a positive change in family dynamics as a result of therapy:

*She’ll join in with the conversations. . . and. . . waffle the whole time. . . she’s not sitting up there sulking anymore. . . (Melanie)*

Similarly, Nancy stated that her son was *‘re-engaged with the family. . . we’ll sit and talk for ages’*. Parents often noted that adolescents’ increased interaction also extended to their social lives:

*She. . . goes to parties now and enjoys herself, whereas before she would have avoided going. (Karen)*

Jane also highlighted the stark contrast to her son’s social behaviour while depressed, stating that *‘there is a huge difference. . . the house is usually full of kids again’.*

#### Lessons learned from CBT

This subtheme depicts what parents thought the adolescent learned through therapy. Many parents said that CBT had helped the adolescent see things from a different perspective. Anne-Marie noted *‘. . .talking about things makes you look at things in a different way because sometimes. . . you don’t really see the big picture’.*

Parents frequently witnessed a shift in their child’s mindset. Mike stated that *‘it [the CBT] was helping her to think. . . more positive about things’*, while Melanie noted *‘in the past. . . she would’ve been harder on herself’*. Parents felt that CBT seemed to make adolescents aware of negative thought patterns. Nancy saw her son’s newfound awareness as stopping him *‘from burying his head in the sand’*, enabling him to change his thoughts and behaviour.

Equipping adolescents with more adaptive skills for coping with uncomfortable situations was seen as an important gain from CBT:

*She [the therapist] was. . . getting her to look at. . . how she copes with things like anxiety situations. (Karen)*

Melanie also observed her daughter’s newly acquired coping mechanisms, noting *‘she seemed to know how to deal. . . she even said. . . when I get upset I know what I need to do now’*.

Additionally, some parents noticed improved self-confidence and self-efficacy, with Jane stating ‘*he can make decisions now. . . he used to have them made for him. . . through his therapy he’s learnt the ways of thinking about things’*.

### Theme 2. Parental perceptions of the therapeutic setting and process

This theme captures how parents experienced factors relating to the therapeutic set-up as impacting on the adolescent’s treatment. It comprises four subthemes: relationship with therapist, management of the therapeutic process, communication with parents, and parental involvement.

#### The importance of a positive adolescent-therapist relationship

A recurring narrative was the perceived importance of a positive relationship between the adolescent and therapist. All parents saw the role of the therapist as key to enabling adolescents to open up:

*It’s always difficult when it’s family. . . you don’t want to upset them. . . whereas when you’re talking to a therapist. . . you can just say exactly what’s on your mind. . . (Lynne)*

Jane stated that her son’s therapist *‘probably knows him more than I do now [laughs]’*, illustrating how close she felt the patient-therapist relationship had become. Parents also pointed out the importance of adolescents being treated as equals. Jane noted *‘the therapist is taking him seriously’*, while Karen stated *‘it wasn’t condescending. . . she [the therapist] was on her level’.*

Most parents who reported that CBT had resulted in little to no improvement attributed this to the lack of connection that they felt their son or daughter had with the therapist. Felicity observed there was *‘no response to what you’ve just said’* and *‘never any eye contact’* and saw this unresponsiveness as negatively impacting the therapeutic relationship. Phil noted *‘the lady she was talking to wasn’t listening. . . if we had got the right person. . . it might have been a different story altogether’*, suggesting the lack of a collaborative relationship with the therapist was viewed as the main reason that therapy was not beneficial and highlighting the pivotal role of the therapist-patient dynamic for a successful outcome.

#### Parents’ trust and confidence in the therapist

This subtheme encompasses parents’ perceptions of the appropriateness of their child’s therapy. While some described therapy as age-appropriate and tailored to their child, others felt it was not personalised:

*She [the therapist] kept on asking. . . about things (my child) didn’t want to talk about. . . I don’t think the therapist was listening to her. . . (Phil)*

Moreover, Phil stated *‘the therapy. . . was. . . something that I could have done myself at home’*, suggesting he saw the therapist as unprofessional and inexperienced.

Some parents questioned the appropriateness of prescribed CBT homework and viewed it as an extra chore for adolescents that added to stress levels. Felicity noted *‘part of what was getting her down was the amount of homework*’, while Jane stated *‘she gives him homework. . . but he’s never done it, ever’.*

Parental accounts about how therapy ended varied widely. Felicity described therapy as ending without warning, saying ‘*. . .it was there one minute and gone the next.’* However, others related how the therapist effectively prepared the adolescent for the end of therapy, for example, by re-iterating warning signs and coping mechanisms:

*She got (name of adolescent) ready for the fact that it was going to end. . . they went through the sort of techniques again. . . and. . . she said you know what to look for now. (Melanie)*

Anne-Marie stated the sessions had *‘done their job’*, indicating her son had got what he needed from therapy and it thus ended at an appropriate time. Others portrayed how their child came to rely less on therapy over time:

*. . .in the beginning he would ask me “when is the next session?” but he doesn’t anymore. . . when. . . the therapist cancelled. . . he wasn’t that bothered. . . (Anne-Marie)*

Parents often viewed the therapy setting as a protected space. Lynne described how the mental health centre, a neutral location, contributed to the feeling of safety, noting *‘the whole environment. . . felt like a safe place where you could talk about anything’.* Jane stated therapy was *‘like a security blanket’* for her son, suggesting that sessions provided a sense of protection.

Moreover, parents seemed to value knowing that therapy was delivered by specialists trained in working with young people:

*That particular floor of that department deals just with children. . . he’s not. . . talking to any old therapist. (Lynne)*

This knowledge seemed to reassure parents that their child was in competent hands.

#### Lack of communication with parents

This subtheme addresses a perceived lack of communication from the clinic or therapist to the parent. While only few parents mentioned the topic, those who did felt strongly about it. Some noted they would have liked to be updated on their child’s progress:

*I didn’t get told anything. . . it would’ve been good to have a briefing on a regular basis. . . (Felicity)*

Another parent felt left in the dark by what she saw as insufficient communication from the clinic:

*I’m there with him. . . every single day and you see him once a month and you know that much and I know nothing. . . (Diane)*

Diane repeatedly expressed that she would have appreciated advice from the clinic on how to support her child during recovery, stating *‘. . .we were supposed to be working together and we were not at all. . .’*, pointing out there was *‘no communication whatsoever. . .’.*

#### Perceived advantages and disadvantages of parental involvement

This subtheme addresses how parents felt their involvement in therapy sessions could hinder or help the therapeutic process. Most seemed to feel their presence would make it more difficult for the adolescent to open up to the therapist, thus choosing not to join their child in CBT sessions:

*I think he would have been much more guarded. . . whereas if I wasn’t there, he would feel more free to talk (Anne-Marie)*

However, some parents viewed their involvement as helpful for both themselves and their child. Susan stated that while her daughter didn’t find the individual sessions *‘useful at all’*, CBT sessions where parents attended gave the adolescent the opportunity ‘*to express herself. . . at home. . . it could get volatile. . . you kind of have to deal with it in the meeting.’ (Susan).*

The family sessions seemed to provide a time and place for adolescents and parents to confront issues together. Nancy described seeing her son *‘in a different light’* through joint sessions, noting *‘in that room it was more like three adults in a room’*. She attributed this dynamic to the therapist’s skill in mediating between parent and adolescent. Karen saw involvement in goal-setting as key to ensuring that adolescent, therapist and parent were on the same page:

*We all had a good understanding of what the goals were. . . you know, singing from the same hymn sheet. (Karen)*

Melanie described finding it important to discuss her own unhelpful behaviours that her daughter found upsetting, which she subsequently worked on changing:

*She [the therapist] would call me in to say. . . (name of adolescent) finds that when you sort of go on at her. . . that makes things worse. . . and I don’t do that now. (Melanie)*

Similarly, Carol stated *‘we would make suggestions of what to do. . . what not to do’*, illustrating that joint parent-adolescent sessions provided a space for reflection on how to make the home environment as supportive as possible.

## Discussion

This study aimed to explore how parents of adolescents diagnosed with depression experienced their child’s CBT. Interviews with parents after therapy had ended generated two main themes. The first theme captured parents’ perceptions of the adolescent’s journey through therapy, and it comprised four subthemes: willingness to engage in therapy, respecting the adolescent’s privacy and offering control, re-emergence of old personality as symptoms recede, and lessons learned from therapy. The second theme captured parents’ perceptions of the therapeutic setting and process, including four subthemes highlighting the importance of a positive adolescent-therapist relationship, parents’ trust and confidence in the therapist, a lack of communication with parents and the perceived advantages and disadvantages of parental involvement in therapy.

Parents viewed the engagement of the adolescent as essential for successful outcomes. It has been reported elsewhere that engagement was a key issue in the IMPACT study; 37% of trial participants dropped out of treatment prematurely (O’Keeffe et al., 2018)., This was for a variety of reasons, classified as – ‘dissatisfied’ dropout, ‘got-what-they-needed’ dropout, and ‘troubled’ dropout (O’Keeffe et al., 2019). Other work has also found that engagement is related to outcomes (Glenn et al., 2013; Weisz & Hawley, 2002). Some parents in our study talked about difficulties getting their child to attend sessions, which they partly attributed to their age. We know that individual factors such as age may impact on readiness to engage in therapy (Chu & Kendall, 2004), possibly due to the increasing need for autonomy as adolescents develop towards adulthood. Involving young people in decision-making processes throughout treatment could be empowering and may help to facilitate successful outcomes (Loh et al., 2007). In our study, parents suggested that encouraging adolescents to take an active role and letting them make decisions promoted their engagement in the therapeutic process. Wilmots et al. (2020), who explored adolescents’ experiences of CBT in the IMPACT trial, found that they similarly experienced therapists who they felt listened to them and involved them in decision-making as empowering and facilitating their recovery. This is also consistent with the wider need to tailor CBT to the age of a child or adolescent (Sauter et al., 2009).

Parents highlighted the importance of the adolescent-therapist relationship with many crediting successful outcomes largely to the quality of this relationship, including a sense of professionalism, trustworthiness, and ability to come alongside the young person. There is evidence that the therapeutic alliance is prospectively associated with outcome in child and adolescent psychological therapy (Karver et al., 2018; Shirk et al., 2011) as well as in adults (e.g., Flückiger et al., 2018), although certain aspects of what parents described (e.g. the importance of the therapist being professional) extend beyond what is usually meant by the ‘therapeutic alliance’. Specific therapist behaviours, such as paying more attention to the adolescent’s experience, have been found in previous studies to lead to greater adolescent involvement in the identification of automatic thoughts, and more extensively exploring the adolescent’s motivation, as well as less strictly structuring sessions was found to contribute to greater task involvement (Jungbluth & Shirk, 2009). Consistent with this, parents in our study emphasised the importance of having a therapist specifically trained to work with adolescents. Some parents viewed treatment as not adequately tailored to their child’s needs and described this as having impacted negatively. Previous research (e.g. Holmbeck et al., 2006) has also suggested that a one-size-fits-all approach makes negative treatment outcomes more likely in adolescents. Some parents in our study felt that the therapist was not listening to the adolescent and that treatment was not personalised. The parental accounts suggest that a flexible CBT which takes the adolescent’s individual needs into consideration can enhance patient engagement and therefore treatment outcomes.

Parents in our study particularly talked about difficulties that they child had in completing therapy homework in between sessions. In CBT completing between-session tasks, or homework, is conceptualised as important because it promotes clients’ autonomy and learning, extends the impact of therapy beyond the treatment session and into ‘real life’, and encourages practice of new skills. Jungbluth and Shirk (2013) found that providing a strong rationale, allocating more time to assigning homework, eliciting reactions to the assignment and foreseeing and troubleshooting possible obstacles can increase homework adherence among depressed adolescents, particularly among those less engaged initially.

Parents often report a major change in their child’s personality during adolescent depression (MacGregor, 1994). Consistent with this, many parents in the present study noted that the adolescent returned to their ‘old self’ once depressive symptoms receded. These findings are in line with Armitage et al. (2020), who reported that parents of depressed adolescents witnessed their children behaving in more familiar ways as they began to recover. Pre-therapy interviews exploring IMPACT participants’ expectations for therapy showed that while some adolescents hoped to re-discover their old self through therapy, others saw it as a chance to develop the self and acquire new capacities (Midgley et al., 2016).

There are different ways in which parents can be involved in therapy. In some studies, for example, the Treatment of Adolescents with Depression Study (TADS) (Wells & Albano, 2005), parent sessions were routinely included as part of CBT. In the CBT arm of the IMPACT study, parent involvement was based on individual need and sometimes entailed parents joining the adolescent in selected therapy sessions. The parents in our study had mixed views about whether their involvement in therapy had been, or would have been, beneficial. Lewinsohn et al. (1990) found that CBT outcomes for adolescent depression were more favourable when therapy included a parent component that entailed separate sessions for parents which provided them with coping skills and encouraged them to reinforce positive change on their child’s part. Dardas et al. (2018) noted that joint sessions, which provided parents and adolescents an opportunity to confront issues together with a therapist present seemed to be even more beneficial than separate parent sessions. In the current study, some parents who attended joint sessions described changing their own behaviours as a result. Integrating psychoeducation for parents more routinely as an adjunct to individual CBT for depression may also give parents practical knowledge to help them support their child during and after therapy, for example, maintaining well-being and recognising early signs of relapse.

## Strengths and limitations

To our knowledge, this is the first study to explore parents’ experiences of CBT for adolescents with depression. Participants were recruited from mainstream publicly funded mental health services and were therefore likely to be fairly representative of the population of adolescents with depression who receive psychological therapy. However, only 16 parent interviews were included and although fathers were actively included in the IMPACT-ME study, most particiapants were mothers. Moreover, the extent of parental involvement in the therapy sessions was quite varied and data on this was not recorded. Future studies could address this by more systematically collecting data on the extent to which parents are involved in CBT for adolescent depression.

We used secondary data in the form of written transcripts. Therefore, it was not possible for the primary researcher to reflect on the potential impact of the interviewer’s biases in the interviews, assess non-verbal communication or probe for further meaning, which may have revealed additional relevant information.

## Clinical implications

Parents emphasised the pivotal role of the adolescent’s willingness to engage in treatment as well as the need to tailor treatment to the age group and the individual at every stage of the therapeutic process. Opening self-referrals to adolescents and offering services that are easily accessible, for example, on school and college campuses, may help ensure that adolescents are willing and able to engage in treatment. Routine clinical practice typically involves an initial assessment of the adolescent’s willingness to engage in treatment, taking maturity and comorbidities into account and adapting goals and treatment accordingly. Our findings highlight the importance of such assessments and adaptations to optimise engagement. The application of motivational interviewing in CBT may further enhance engagement and potentially increase rates of treatment response (Zuckoff et al., 2008).

Parents also underscored the value of the adolescent-therapist relationship. Some therapists were perceived as not listening to the young person or meeting their needs. Although not experienced by most parents, these aspects are cause for concern. Therapists should routinely seek feedback about both progress and process at every session. A range of simple and brief patient reported outcome measures (PROMS) is freely available and recommended as part of routine clinical care in child and adolescent mental health services (Child Outcomes Research Consortium [CORC], 2020). Our results suggest that better implementation of PROMS into clinical care, with specific attendance to early signs of non-engagement, could help improve the experience of therapy for young people and their parents and potentially improve treatment outcomes.

While it is common practice for parental involvement to be agreed upon by the adolescent, parent and therapist prior to treatment, the role of the parent can assume different forms in CBT. It may be beneficial for parent involvement to go beyond attending therapy sessions. For example, parents may act as facilitators (Stallard, 2002) by encouraging their child to apply their newly acquired coping skills at home. Moreover, therapists may treat parents as co-clients (Stallard, 2002), providing them with psychoeducation on depression and addressing helpful and hindering parental behaviours. Communication with parents who were not involved in treatment sessions was often minimal. Many parents would welcome more frequent communication with the therapist even if they are not directly involved in therapy. Future research to examine how best to involve parents is needed.

## Conclusion

Parents perceived the willingness of adolescents to engage in treatment and a strong adolescent-therapist relationship as crucial when treating adolescent depression. This requires a flexible therapeutic approach. Parents also highlighted the importance of adolescents, therapists and, as appropriate, parents working together to ensure that the adolescent’s individual needs are met. The insights gained from parents’ unique perspectives confirm the foundations of what is viewed as good clinical practice for treating adolescent depression.
